# Harnessing LSTM and XGBoost algorithms for storm prediction

**DOI:** 10.1038/s41598-024-62182-0

**Published:** 2024-05-18

**Authors:** Ayyoub Frifra, Mohamed Maanan, Mehdi Maanan, Hassan Rhinane

**Affiliations:** 1 UMR 6554 CNRS LETG-Nantes Laboratory, Institute of Geography and Planning, Nantes University, 44312 Nantes, France; 2 Geosciences Laboratory, Faculty of Sciences Ain Chock, Hassan II University of Casablanca, 20100 Casablanca, Morocco

**Keywords:** Physical oceanography, Natural hazards, Environmental impact

## Abstract

Storms can cause significant damage, severe social disturbance and loss of human life, but predicting them is challenging due to their infrequent occurrence. To overcome this problem, a novel deep learning and machine learning approach based on long short-term memory (LSTM) and Extreme Gradient Boosting (XGBoost) was applied to predict storm characteristics and occurrence in Western France. A combination of data from buoys and a storm database between 1996 and 2020 was processed for model training and testing. The models were trained and validated with the dataset from January 1996 to December 2015 and the trained models were then used to predict storm characteristics and occurrence from January 2016 to December 2020. The LSTM model used to predict storm characteristics showed great accuracy in forecasting temperature and pressure, with challenges observed in capturing extreme values for wave height and wind speed. The trained XGBoost model, on the other hand, performed extremely well in predicting storm occurrence. The methodology adopted can help reduce the impact of storms on humans and objects.

## Introduction

Storms and extreme extratropical cyclones represent a significant risk to human life and the environment^1–5^. They are the primary natural hazard affecting Western and Central Europe and frequently have a devastating impact on human activity and infrastructure^6–8^. Most countries have experienced a rise in economic losses due to storms^9,10^. However, extratropical cyclones are also necessary as they are responsible for the majority of precipitation in large parts of the world; over 70% of the total precipitation in Western and Central Europe and much of North America, for example, is associated with the passage of extratropical cyclones^11^.

In Western France, several storms have resulted in significant human and material loss. On December 26 and 28, 1999, Storms Lothar and Martin caused considerable damage and many fatalities; 92 people were killed, and 3.5 million households were left without electricity for several weeks^12^. In addition, much damage was done to forests and buildings, with roofs either totally or partially blown off^13^. Another tragic event, Storm Xynthia, occurred on February 28, 2010, leading to heavy loss of life and devastating damage to infrastructure. The combination of the storm and a high tide caused several sea walls to rupture, leading to extensive flooding in low-lying coastal zones, more than two billion euros’ worth of damage, and 47 fatalities^14^.

The increased likelihood of extreme weather events and their risk^15^ have received considerable attention in recent decades^16–18^. Predicting these events is highly challenging; Ensemble Prediction Systems (EPS) can generate short-term forecasts, for example the EPS at the European Centre for Medium-Range Weather Forecast (ECMWF) which predicted storms Anatol, Lothar, and Martin in December 1999^19^. Predicting storms with lead times of more than a couple of weeks can be achieved with seasonal forecast systems, such as the DEMETER (Development of a European Multimodel Ensemble System for Seasonal-to-Interannual Prediction) and ENSEMBLES (ENSEMBLE-based predictions of climate changes and their impacts) projects, which produce reliable forecasts of storms over the North Atlantic and Europe^20^, and modern seasonal forecast systems from the ECMWF and the Met Office Hadley Centre, used to forecast the frequency of storms over the Northern Hemisphere^21^. Storms can also be predicted by forecasting a specific variable associated with storm events, as is the case with the local sea wave model of the German Weather Service, which can predict severe winter storms connected with extraordinarily high waves^22^.

The use of machine learning (ML) and deep learning (DL) models is increasingly common in the field of extreme weather events. Their main function is to predict storm characteristics; artificial neural networks combined with wavelet analysis, for example, are used to predict extreme wave height during storms^23^. Evaluation of the results has indicated that ML and DL solutions provide better results than traditional methods in terms of computational expense and accuracy of predictions^24^, and can serve as an alternative tool to conventional models for forecasting storms^25^.

In recent years, recurrent neural networks (RNNs) have attracted considerable attention as a time-series prediction tool^26,27^. Despite the ability of RNNs to understand and capture short-term dependencies, they experience difficulty with long-term dependencies due to the vanishing gradient problem^28^; the backpropagation error disappears or vanishes in the earlier inputs after propagating through several steps. Consequently, the standard RNN cannot learn effectively, and the information from earlier stages is ignored in the prediction task^29^. To overcome this drawback and improve the memorization of RNNs, a new model, the LSTM algorithm, was developed as an extension to RNNs, and can learn long-term dependencies from time series data and resolve the vanishing gradient problem^30^. The LSTM algorithm has performed very well with a large variety of issues. It has successfully resolved many sequence learning problems and time-series predictions, such as speech recognition^31^, machine translation^32^, and wind speed prediction^33,34^. The LSTM algorithm has also been used for predicting extreme weather events, for example the storm waves induced by two winter storms in the US North Atlantic region^25^. The model was trained using buoy measurements and the findings demonstrated that the LSTM model can accurately predict the characteristics associated with storm events^25^. In a second study, the LSTM model proved its efficiency in predicting storm characteristics by forecasting significant wave height and wave period at storm peaks, based on data from two offshore buoys. The results demonstrate its ability to capture all major wave events, including storm peaks, and serve as a tool for early storm warning^24^.

XGBoost is another robust ML algorithm widely used in many applications and given rave reviews by ML practitioners^35^. This algorithm is a scalable ML-based technique for the tree boosting method introduced by Friedman^36^. XGBoost, developed by Chen and Guestrin^37^, has proven its efficiency in various classification and regression time series problems, such as rare-event classification; Ranjan et al. used highly imbalanced data from a pulp and paper mill as input for XGBoost to forecast paper break events^38^. XGBoost is a flexible ML method capable of dealing with the non-linearity of time series with its efficient self-learning ability^39^. It performs extremely well in time series prediction, with adequate computing time and memory resource usage^40^. The XGBoost model has also been used to predict wind-wave conditions at storm peaks based on hourly wind, significant wave height, and peak wave period observations at buoy stations. The findings indicated the ability of XGboost to predict wave dynamics under extreme conditions^24^.

Prior research has investigated the use of LSTM and XGBoost models in storm prediction, as shown by studies conducted by Hu et al.^24^ and Ian et al.^41^. Nevertheless, this study presents an innovative technique that deviates from current approaches. Prior investigations have mostly concentrated on using LSTM and XGBoost independently and comparing their performance in storm surge prediction, usually considering it as a regression problem. In contrast, the proposed approach in this paper cooperatively combines both models to forecast storm characteristics and occurrence. Specifically, LSTM was used for regression-based prediction of various storm characteristics, while XGBoost was used for classifying the days of storm occurrence. This integrated approach allows for a more thorough investigation of storm prediction. In addition, previous research has often focused on forecasting a limited number of storm variables, such as wave height or water level. This paper expands the forecast scope to include a wider variety of storm variables, such as wave height, wave period, wind speed, temperature, pressure, and humidity. In summary, this study builds upon existing literature by including a broader spectrum of storm characteristics and using an innovative and comprehensive method for forecasting storms, which will help enhance preparation and mitigation strategies.

With the main goal of providing a new data-driven approach to forecasting storm occurrence and characteristics, this article also aims to investigate the effectiveness of two of the best models currently available, XGBoost and LSTM, in terms of providing valuable predictions of rare events, such as storms. It then presents first the methods, followed by the results, discussion, and then conclusions.

## Methods

The research roadmap involved two methods: LSTM and XGBoost. The first is designed to predict the different characteristics of storms, and the second to predict the occurrence of storms based on their characteristics.

### Study site and data

**Figure 1:**
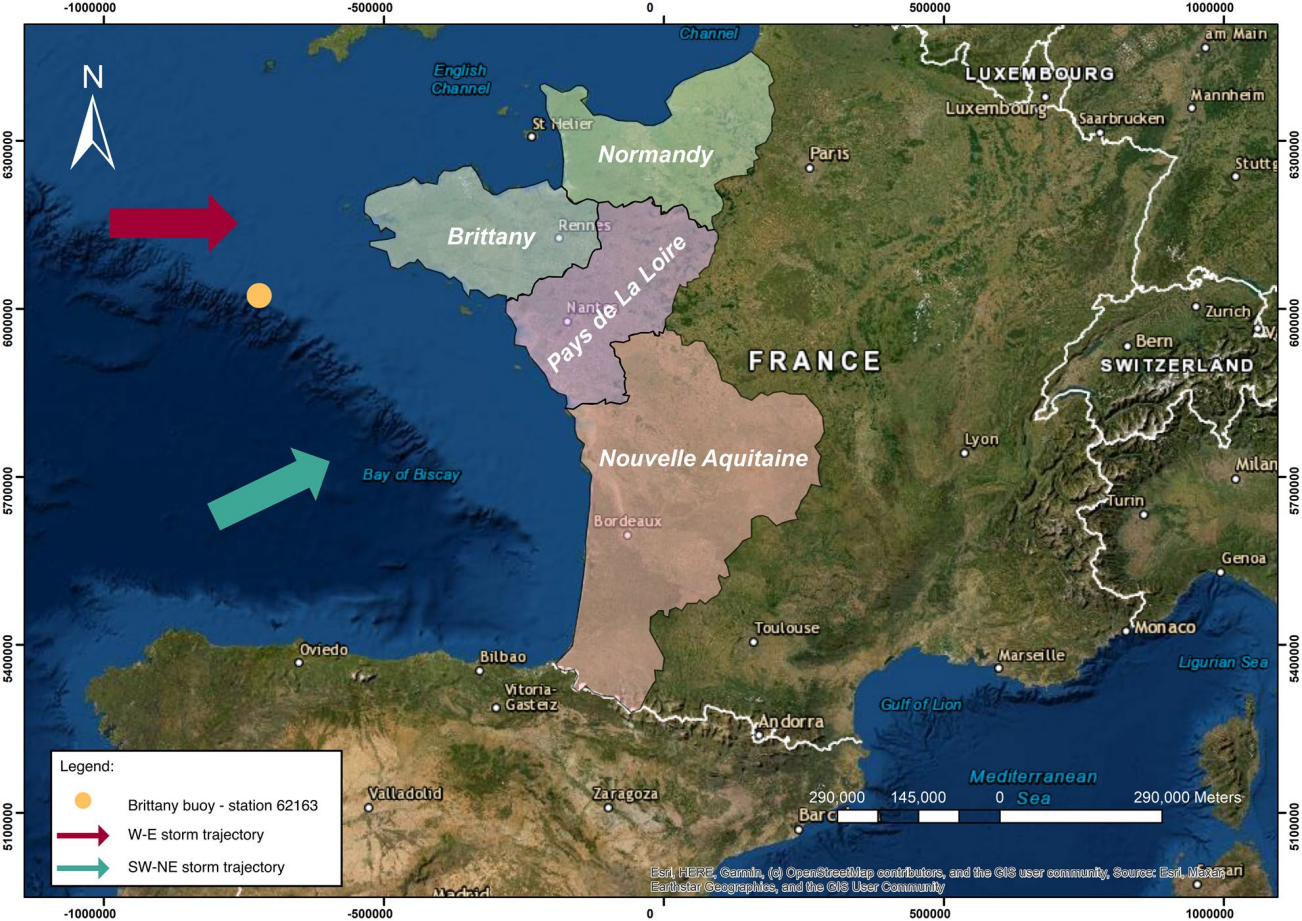
Location of the study area.

The site for this study was the western coast of France, comprising four regions: Normandy, Brittany, Pays de la Loire, and Nouvelle-Aquitaine (Fig. 1). These regions have experienced unexpected storms, resulting in many human and material losses^12,14^, highlighting the need for historical reconstruction of past storms. The study area has therefore been the subject of numerous studies to identify past storm dynamics, their relation to climatological mechanisms, their trajectories, and the spatialization of damage^42,43^. These studies have identified two main storm paths: The first estimated from west to east and impacting a restricted area of the French Atlantic coast, the second more extensive, with a southwest-to-northeast trajectory affecting a larger section of the coast^42^. These results concur with the classification method known as Dreveton, which proposes a classification of storms based on the origin of the associated depression, either Atlantic or Mediterranean; those associated with low-pressure systems over the Atlantic are more frequent than those from the Mediterranean^44^. Two classes of storm directly affect the Atlantic coast. The first relates to storms generated from depressions over the British Isles. The storm tracks, therefore, pass through Brittany then move up towards northern France, affecting either the north of France or the entire country. The other storm class relates to those generated from depressions over the Bay of Biscay. The trajectory of this second category of storms moves inland via the Pays de la Loire—Poitou—Charentes and continues towards the east of France and then Germany, affecting the northern part of France or the whole country. The studies conducted by Castelle et al.^45,46^ have greatly contributed to enhancing the comprehension of storm impacts and creating predictive tools for reducing coastal hazards in the study area. For example, their study of the effects of intense winter storms in 2013–2014 provides insights on the erosion patterns and the development of megacusp embayments^45^. This research focuses on the correlation between storm wave attributes, such as wave height, duration, and angle of incidence, and the consequent patterns of erosion^45^. In addition, Castelle et al.^46^ developed a new climate index based on sea level pressure, termed the Western Europe Pressure Anomaly (WEPA) index, which accurately accounts for the variations in winter wave height throughout the Western European coast. The WEPA index outperforms other prominent atmospheric modes in explaining winter wave variability and excels at capturing extreme wave height events, as demonstrated by its performance during the severe 2013–2014 winter storms that affected the study area^46^. Moreover, Castelle et al.^47^ conducted a study on the changes in winter-mean wave height, variability, and periodicity in the northeast Atlantic from 1949 to 2017, and examined their connections with climate indicators. Their research highlighted the growing patterns in the height, variability, and regularity of winter waves. These patterns are mainly influenced by climate indices like the North Atlantic Oscillation (NAO) and WEPA. As the positivity and variability of the WEPA and NAO increase, the occurrence of extreme winter-mean wave heights becomes more common^47^, which makes these indices valuable predictors in forecasting coastal hazards.

This study combines data from a storm database and offshore buoy data from 1996 to 2020. The Meteo France website provided the storm database, containing all the storm events in the study area (http://tempetes.meteo.fr/spip.php?rubrique6), and the historical measurements from the Brittany Buoy—station 62,163, with hourly records of wave height, wave period, wind speed, temperature, pressure, and humidity from January 1, 1996 to December 31, 2020 (https://donneespubliques.meteofrance.fr/?fond=produit&id_produit=95&id_rubrique=32). These hourly records were transformed into daily values by calculating the average value for each day and adding this to the storm names and occurrences extracted from the storm database. Storm occurrence is defined as days with wind speed values ranking above the 98th percentile of historical wind speed measurements from buoy data and coinciding with the days identified in the storm database as having storm events. Using this definition of storm occurrence, we were able to identify all the storm events in the study area since 1996. The occurrence of storms followed a binomial probability distribution, where the value 1 represented the occurrence of a storm, and the value 0 represented no storm. The combination of buoy data and storm database provided multivariate time series data (Table 1) representing a day-by-day record of historical weather and marine data and the precise days on which storms occurred, which could be used to predict the occurrence of storms and their different characteristics.

**Table 1:** Part of the dataset used for the prediction at Brittany Buoy—station 62,163.

Date	Storm name	Storm occurrence	Wave height (m)	Wave period (s)	Wind speed (m/s)	Temperature (K)	Pressure (Pa)	Humidity (%)
01-01-1996	–	0	4.336	9.494	6.931	285.249	99,436.485	87.471
02-01-1996	–	0	3.452	8.236	7.457	286.219	101,116.521	88.826
…	…	…	…	…	…	…	…	…
26-12-1999	Lothar	1	7.795	9.757	23.95	286.046	97,000	81.588
27-12-1999	Martin	1	6.852	9.828	22.853	297.942	96,480	83.533
28-12-1999	–	0	5.404	8.875	9.315	285.420	101,280	70.210
…	…	…	…	…	…	…	…	…
30-12-2020	-	0	3.45	7	9.808	283.770	101,017.1	79.291
31-12-2020	-	0	2.654	6.041	9.704	282.508	101,144.6	67.833

### Prediction of storm characteristics

The dataset was fed into a multivariate LSTM model to predict the values of the six storm characteristics at once. The following steps were taken to prepare the data for implementing the algorithm: first, any missing values for each attribute were replaced with the median value for all the known values of the attribute. This approach is more reliable than mean imputation when the data includes outliers or is skewed since the median is less impacted by extreme values. The LSTM model was then constructed in the Keras framework, the input to the LSTM consisting of a time series vector x for T time steps in the series.

$$x=\left[x\left(1\right), x\left(2\right), \dots , x\left(T\right)\right]$$

Each input vector x(t) contained the six characteristics:

$$x\left(t\right)=[Temperature\left(t\right), Humidity\left(t\right), Wind speed\left(t\right), Pressure\left(t\right), Wave height\left(t\right), Wave period(t)]$$

These input sequences were generated by moving a time window through the dataset, each time capturing a sequence of T input vectors. An input of 30-time steps was favored during hyperparameter optimization. Consequently, the characteristics from time (T-30) to (T-1) were used as features to predict the corresponding characteristics y at time T.

$$y\left(T\right)=\left[Temperature\left(T\right), Humidity\left(T\right), Wind speed\left(T\right), Pressure\left(T\right), Wave height\left(T\right), Wave period\left(T\right)\right].$$

To train and test the LSTM model, the time series data were split into training and test sets at a ratio of 80% to 20%. Each division was given a continuous period of the time series to preserve the serial correlation between successive observations. This yielded a training dataset with an input size of $\left[nx, T, v\right]=[7281, 30, 6]$, where $nx$ is the number of training sequences of length T and $v$ is the number of variables, and a test dataset with an input size of [1821, 30, 6]. The training dataset only was used to select the optimal hyperparameters, and the test partition, used to test how well the model generalizes to unseen conditions, was set aside to avoid information leakage. After splitting the data, the standard score normalization (Z-Score) method was applied to normalize the input variables. The values for an attribute were normalized by subtracting the mean for each value in the distribution and dividing the result by the standard deviation^48^. Normalization helps speed up the learning phase and prevents initially large-range attributes from outweighing initially smaller ones^48^.

Optimization of the LSTM model required decisions on a combination of large hyperparameters, such as the number of neurons, number of layers, batch size, and learning rate. A scalable hyperparameter optimization framework called Keras Tuner, based on the Bayesian optimization algorithm, was therefore applied to find the best hyperparameter values. To optimize the parameters, this neural network was trained using cross-validation and by setting mean absolute error (MAE) as a performance evaluator. The TimeSeriesSplit technique was used to apply the cross-validation. This strategy is especially appropriate for our investigation since it preserves the temporal order of the data, which is critical for the predictive accuracy of the LSTM model. The model was therefore trained and validated in three splits, which allowed us to systematically analyze and enhance the model's performance by modifying hyperparameters using the Bayesian optimization strategy. Table 2 lists the details of hyperparameter value ranges and choices, and the following paragraph covers each hyperparameter tuned using the Keras Tuner.

**Table 2:** Hyperparameters optimization of the LSTM and XGBoost models.

	Hyperparameters	Value Ranges			Choices	Value
		Min	Max	Step		
LSTM	Number of layers	1	4	–	–	–
	LSTM layers units	32	512	32	–	–
	Dropout	0	0.5	0.1	–	–
	Learning rate	–	–	–	0.01, 0.001, 0.0001	–
	Batch size	–	–	–	16, 32, 64, 128	–
	Optimizer	–	–	–	–	Adam
	Objective	–	–	–	–	val_mean_absolute_error
	Max trials	–	–	–	–	20
	Executions per trial	–	–	–	–	2
XGBoost	learning_rate	–	–	–	0.0001, 0.001, 0.01, 0.1	–
	max_depth	–	–	–	3, 4, 5, 6, 7, 8, 9, 10, 11, 12	–
	gamma	–	–	–	0, 0.1, 0.2, 0.3, 0.4	–
	colsample_bytree	–	–	–	0.3, 0.4, 0.5, 0.6, 0.7, 0.8, 0.9	–
	reg_alpha	–	–	–	0.00001, 0.01, 0.1, 1, 10, 100	–
	reg_lambda	–	–	–	0.00001, 0.01, 0.1, 1, 10, 100	–

Twenty different model configurations were tested to find the optimum set of hyperparameters. For each hyperparameter combination, the LSTM model was trained for a maximum of 100 epochs, and the epoch corresponding to the minimum validation set MAE was recorded. The Google Colab A100 GPU system was used to run the entire model training. The best-proposed model by the Keras Tuner applied three layers. The minimum and maximum numbers of neurons at each hidden layer were set as 32 and 512 respectively. The dropout layer, used to prevent overfitting by eliminating certain connections between neurons in each iteration^49^, was defined within the range of 0 to 0.5. The output layer was then linked to a dense layer with six output neurons and the activation function was set to Linear. The network was compiled with a MAE loss function and Adam optimizer to update the weight of each layer after each iteration. The learning rate, which was set using Keras Tuner to one of three values (0.01, 0.001, or 0.0001), determined the size of the step at each iteration of the optimization method^49^. Four other value choices (16, 32, 64, 128) were set for the batch size, representing the number of sub-samples the network used to update the weights^49^. Proceeding through the search space, the combination with the lowest validation set MAE was chosen as the optimum set of hyperparameters (Table 3).

**Table 3:** Optimal set of hyperparameters found for LSTM and XGBoost.

	Hyperparameters	Best hyperparameter values
LSTM	Number of layers	3
	LSTM layer 1 unit	192
	LSTM layer 2 units	288
	LSTM layer 3 units	32
	Dropout	0.2
	Activation function for output layer	linear
	Learning rate	0.01
	Batch size	32
	Optimizer	Adam
	Time steps	30
XGBoost	learning_rate	0.1
	max_depth	12
	gamma	0
	colsample_bytree	0.4
	reg_alpha	0.00001
	reg_lambda	0.01

**Figure 2:**
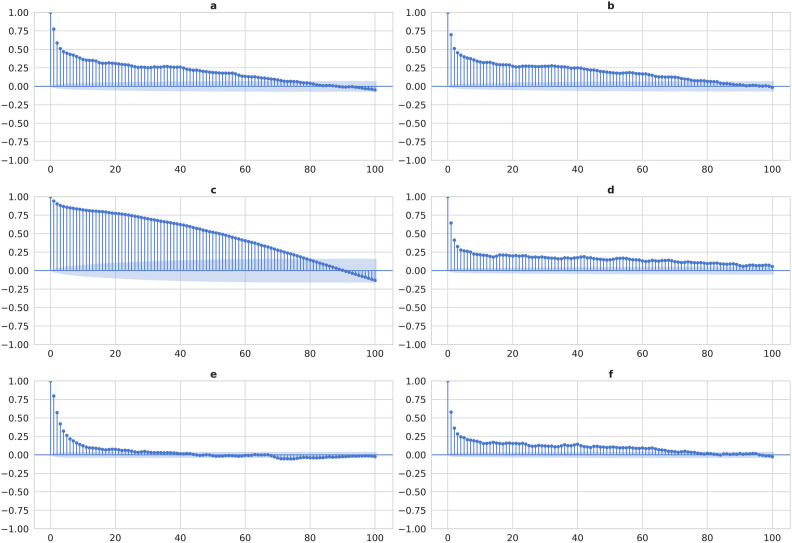
Autocorrelation Analysis (**a**) Autocorrelation of Wave Height (**b**) Autocorrelation of Wave Period (**c**) Autocorrelation of Temperature (**d**) Autocorrelation of Humidity (**e**) Autocorrelation of Pressure (**f**) Autocorrelation of Wind Speed.

A comprehensive analysis of autocorrelation functions (ACF), together with a trial-and-error process, was used to identify the ideal input time steps for improving the model's performance. The use of the ACF on the daily dataset demonstrates how each attribute corresponds with its historical observations. As seen in Fig. 2, the autocorrelation plots for pressure and wind speed indicate a shorter memory effect, with substantial correlations vanishing rapidly. In contrast, humidity has longer-term meaningful associations that stay generally steady over time. Temperature, wave period, and wave height show more gradual decay, indicating a longer memory effect. Based on the findings from the ACF analysis, a sequence length that captures the memory of the most persistent variable while still giving useful information for variables with shorter memories should be selected. As a result, a sequence length of 20-time steps was chosen as an ideal compromise. This input sequence was thought sufficient to capture the longer-term dependency found in temperature and wave-related variables, while also catching essential shorter-term information for pressure and wind speed. The model's performance was then examined by training and validating the LSTM model using cross-validation with the initial sequence length selected based on the ACF results. The sequence length was further optimized by gradually decreasing and increasing the number of lags used to anticipate future values until the optimal sequence length was determined. This iterative procedure resulted in the discovery of an optimal input sequence of 30-time steps, allowing the LSTM model to achieve higher performance.

MAE, mean absolute percentage error (MAPE), root mean square error (RMSE), and coefficient of determination (R^2^) were used to evaluate the performance of the LSTM model. These evaluation metrics are defined as follows:

1 $$MAE=\frac{1}{n}\sum_{i = 1}^{n}\left| {y}_{i}-{\widehat{y}}_{i}\right|$$

2 $$MAPE= \frac{100}{n} {\sum }_{i=1}^{n}\left|\frac{{y}_{i - {\widehat{y}}_{i}}}{{y}_{i}}\right|$$

3 $$RMSE = \sqrt {\frac{1}{n}\mathop \sum \limits_{i = 1}^{n } (y_{i} - \hat{y}_{i} )^{2} }$$

4 $${R}^{2}=1- \frac{{\sum }_{i=1}^{n}{({y}_{i - }{\widehat{y}}_{i})}^{2}}{{\sum }_{i=1}^{n}{({y}_{i -} \overline{Y })}^{2}}$$

where $n$ is the number of samples, ${y}_{i}$ the real value, ${\widehat{y}}_{i}$ the predicted value, and $\overline{Y }$ is the mean of the observed values. The best performance is achieved when the values of MAE, MAPE, and RMSE are close to 0 and the value of R^2^ is close to 1.

The Pearson correlation coefficient (r) was also used to assess the performance of the LSTM model, specifically the strength of the linear association between the observed and predicted values. The definition of this measure is as follows:

5 $$r= \frac{{\sum }_{i=1}^{n}({x}_{i}- \overline{x })({y}_{i}- \overline{y })}{\sqrt{{{\sum }_{i=1}^{n}({x}_{i}- \overline{x })}^{2}{\sum }_{i=1}^{n}{({y}_{i}- \overline{y })}^{2}}}$$

where ${x}_{i}$ are the observed values, ${y}_{i}$ are the predicted values, $\overline{x }$ and $\overline{y }$ are the means of the observed and predicted values, respectively, and $n$ is the number of samples.

### Prediction of storms

The objective of the second part was to predict the occurrence of storms based on their characteristics. To achieve this, an XGBoost binary classifier was developed using the XGBoost library. The XGBoost model used the six characteristics as input ${X}_{i}$, and storm occurrence was used as model output ${y}_{i}$, given a binary class label ${y}_{i }\in \{\mathrm{0,1}\}$ , indicating the absence or occurrence of a storm, respectively (Table 1). The median value of each attribute was used to fill in the missing values of the independent variables ${X}_{i}$. The dataset was split into training and testing sets at the ratio of 80 to 20. The 20% testing data (unseen data) corresponding to the period from January 2016 to December 2020 was set aside to avoid information leakage. The training dataset from January 1996 to December 2015 only was used for tuning the hyperparameters. The preprocessing phase of normalizing independent variables ${X}_{i}$ was carried out using the method described in the section ‘Prediction of storm characteristics’. This section applied Bayesian optimization using the Hyperopt library in Python to find the best combination of hyperparameters. Bayesian optimization using Hyperopt is an effective method for Hyperparameter optimization of the XGBoost algorithm. It performs better than other widely-used approaches such as grid search and random Search^50^.

The XGBoost model was trained using stratified k-fold cross-validation with K = 3. Stratified k-fold is highly appropriate for imbalanced data, as in our study, as it helps keep the class ratio in the folds the same as the training dataset^51^. The training dataset was divided into three subintervals—two for training and one for evaluating. Six hyperparameters were optimized, namely Learning rate (eta), Maximum Tree Depth, Gamma, Column samples by a tree, Alpha, and Lambda, and the search space defined is shown in Table 2. Fifty different combinations were tested to find the optimum set of hyperparameters. For each combination, recall was set as the evaluation metric for cross-validation. The combination at which the highest recall value was found is identified as the best set of hyperparameters (Table 3). After identifying the best hyperparameters, XGBoost was trained with the entire training dataset and tested using the unseen dataset. The Google Colab A100 GPU system was used to run the hyperparameter optimization and model training.

Due to the low frequency of storm occurrence, the following performance measures were used to evaluate the accuracy of the model: Recall, specificity, false positive rate (FPR), and false negative rate (FNR). These assessment metrics are not sensitive to imbalanced data^52^ and can be calculated using the following equations:

6 $$\mathrm{Recall }({\text{Sensitivity}})=\mathrm{ True positive rate}= \frac{TP}{TP+FN}$$

7 $$\mathrm{Specificity }=\mathrm{ True negative rate}= \frac{TN}{TN+FP}$$

8 $${\text{FPR}}= \frac{FP}{FP+TN}$$

9 $${\text{FNR}}= \frac{FN}{FN+TP}$$

where:

- $TP$: true positives, where the model predicted samples correctly as positive. In this case, the storms were classified as ‘storm’.
- $TN$: true negatives, where the model predicted samples correctly as negative (no storms predicted as ‘no storm’).
- $FP$: false positives, where the model wrongly predicted samples as positive (no storms predicted as ‘storm’).
- $FN$: false negatives, where the model wrongly predicted samples as negative (storms predicted as ‘no storm’).

The performance of the model was also measured using ROC (receiver operating characteristics) curve. ROC curve is a two-dimensional graph where the x-axis is the FPR and the y-axis the TPR (true positive rate), and it is generated by changing the threshold on the confidence score. The ROC curve is not sensitive to imbalanced data and can illustrate the diagnostic capability of a binary classifier^52^. The area under the ROC curve (AUC) metric is used to assess the performance of the XGBoost model since there is no scalar value representing the expected performance in the ROC curve. The AUC metric ranges from 0 to 1, and a perfect model will have an AUC of 1.

## Results

### LSTM results

**Figure 3:**
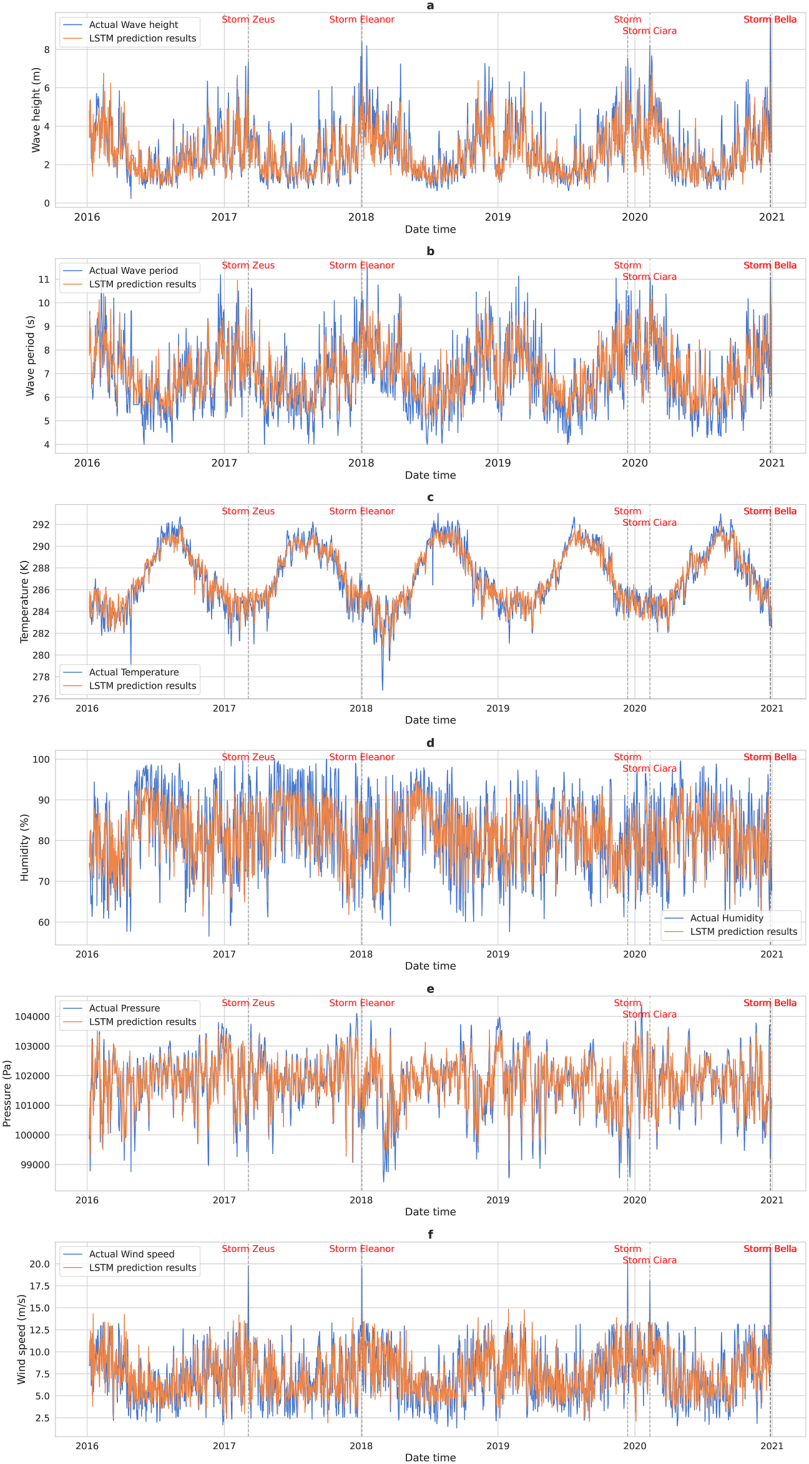
Prediction outcomes of the LSTM model for the unseen test partition (**a**) Wave height prediction results (**b**) Wave period prediction results (**c**) Temperature prediction results (**d**) Humidity prediction results (**e**) Pressure prediction results (**f**) Wind speed prediction results..

The trained LSTM model was used to predict storm characteristics for the unseen test period from January 2016 to December 2020. The prediction errors are summarized in Table 4. Figure 3 shows the prediction outcomes of the LSTM model. The blue curve is the actual values and the orange curve is the forecast results. The figure shows that the model provides a good representation of the observed values for most of the time period as the actual and predicted curves roughly correspond.

**Table 4:** Evaluation metrics of the LSTM and XGBoost models.

		MAE	RMSE	MAPE	R^2^	r
LSTM	Wave height	0.6271	0.8617	24.27%	0.6083	0.7914
	Wave period	0.6439	0.8189	9.69%	0.6522	0.8141
	Temperature	0.7574	0.9881	0.26%	0.8753	0.9385
	Humidity	6.0851	7.6008	7.61%	0.3272	0.5843
	Pressure	467.2703	630.4106	0.46%	0.5536	0.7495
	Wind speed	2.0061	2.5666	32.76%	0.2337	0.5482
XGBoost	Recall	Specificity	FPR	FNR	**–**	**–**
	1	1	0	0	–	–

The LSTM model demonstrated impressive accuracy in temperature predicting, as shown by a low MAE of 0.7574 K, an RMSE of 0.9881 K, and a MAPE of 0.26%. In addition, the model's R^2^ score of 0.8753 and Pearson Correlation Coefficient of 0.9385 indicate that it effectively captures a substantial amount of the temperature data's variability and exhibits a robust linear association with the actual values.

The performance of the model for humidity forecasts was less accurate, with MAE and RMSE values of 6.0851% and 7.6008%, respectively. The moderate value of the R^2^ score (0.3272) and the comparatively high MAPE value (7.61%) show the model's limited skill in capturing the variation in humidity. The Pearson Correlation Coefficient of 0.5843 suggests a moderate linear connection, however with much opportunity for improvement.

The LSTM model faced a larger difficulty in predicting wind speed, as evidenced by the high MAPE of 32.76%, MAE of 2.0061 m/s and RMSE of 2.5666 m/s. In addition, the low R^2^ score (0.2337) and modest Pearson Correlation Coefficient (0.5482) demonstrate the model's limitations in predicting wind speed. The low performance of the LSTM for wind speed prediction might be due to the complex and varied nature of wind behavior, and the presence of very extreme values (outliers) at storm events.

The model's performance for pressure prediction was relatively good, with MAPE of 0.46% indicating that the model's predictions closely match the actual pressure levels. The R^2^ score of 0.5536 and the Pearson Correlation Coefficient of 0.7495 indicate that the model is capable of capturing the variability in pressure.

The predictions of wave conditions (wave height and period) by the LSTM model were reasonable, with MAE values of 0.6271 m and 0.6439 s, respectively, and RMSE values of 0.8617 m and 0.8189 s, respectively. However, the high MAPE of 24.27% in wave height shows a considerable relative error, demonstrating the underestimation of the model in wave height prediction during storm events. The R^2^ scores of 0.6083 and 0.6522 for wave height and period, together with Pearson Correlation Coefficients of 0.7914 and 0.8141, imply a high predictive association with the actual values.

The model’s forecasts at storm events for temperature and pressure were particularly accurate, whereas humidity revealed inconsistencies when compared to the observed values. Predictions of wave-related variables were consistent but underlined the necessity for refinement, specifically for wave height, which showed a tendency to be underpredicted during storm events. An even bigger underprediction can be seen towards the extreme values of wind speed during storm events.

The low frequency of storm occurrence can explain these findings; storm events represent only 0.7% of the total data used in this study. Most of the available data for training therefore covers lower wave height and wind speed values, which correspond to non-storm events. More storm observations are required in the training dataset to improve further the accuracy of the model in predicting storm characteristics.

In conclusion, the LSTM model exhibits proficiency in predicting temperature and pressure characteristics of storms with high accuracy. The model provides a robust foundation for temperature and pressure forecasts, while the prediction of humidity, wind speed, and wave characteristics shows potential but requires further optimization.

### XGBoost results

**Figure 4:**
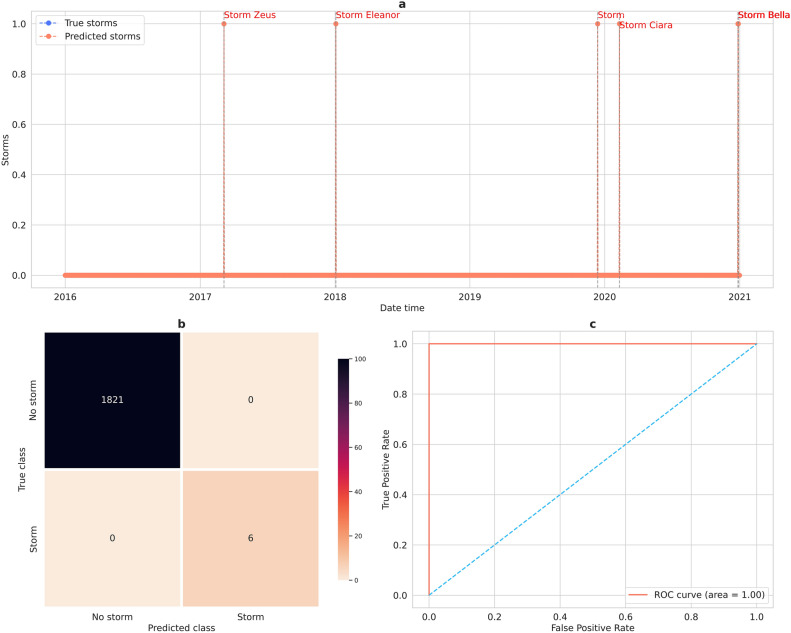
Prediction of storms using XGBoost model (**a**) Storm occurrence prediction results (**b**) Confusion matrix of the XGBoost model (**c**) ROC curve of the XGBoost model.

The trained XGBoost model was used to predict storm occurrence for the unseen test period of January 2016—December 2020. There were five storms in this period: Zeus on March 6, 2017, Eleanor on January 3, 2018, storm on December 13, 2019, Ciara on February 10, 2020, and Bella, a two-day storm on December 27 and 28, 2020. There are two classes: Storm and No-storm. The Storm class is positive, and the No-storm class is negative. Figure 4a shows the prediction of storm occurrence; predicted storms are represented by light orange points and actual storms by light blue points. As shown in the figure, the predicted storms concur precisely with the actual storms; the model predicted the occurrence of all the storms. The confusion matrix of the XGBoost model is given in Fig. 4b. Six samples are correctly classified as Storm and 1821 correctly classified as No-storm; the respective TN, TP, FN, and FP values are therefore 1821, 6, 0, and 0. The classification metrics are given in Table 4. The proposed XGBoost classifier performed very well in predicting storm occurrence. Recall and specificity values, which represent the correctly predicted samples, are equal to 1, while the FPR and FNR, which represent the incorrectly predicted samples, are equal to 0. Figure 4c shows the AUC value of the model. As shown, the model has an AUC of 1, which implies higher accuracy in predicting storm occurrence.

## Discussion

This study applied a new data-driven approach based on LSTM and XGBoost to forecast storm characteristics and occurrence in Western France. There were two main elements to the study. First, comprehensive research into storm prediction using six different storm attributes and the days on which they occurred. Second, exploring the efficiency of the ML and DL methods in capturing rare events such as storms. Experiments were conducted to build a multivariate LSTM model to predict storm characteristics. The LSTM model was evaluated using the unseen test period data from January 2016 to December 2020. In a second step, an XGBoost binary classifier was developed to predict the occurrence of storms based on their characteristics, and data from January 2016 to December 2020 was used for model evaluation. The following key points elaborate on the outcome of this study.

The LSTM model was found to perform well in predicting the six variables (wave height, wave period, wind speed, temperature, humidity, and pressure) over all the unseen test period. The visual comparison of forecasted and observed values demonstrated that the model typically produced a decent representation of actual values. During storm events, the accuracy of the LSTM model in forecasting storm features fluctuated significantly. It excelled in anticipating temperature and pressure, displaying high accuracy. However, it proved less effective in forecasting humidity and notably underpredicted wave-related variables and wind speed during storms. This tendency to underestimate wave height and wind speed can be explained by the limited number of extreme wave heights and wind speeds available for model training. Comparable results were found by Hu, Haoguo, et al. for predicting wave height during storm events^24^. The models tended to underestimate extreme wave height and perform extremely well for regular events^24^. To overcome this issue, Dixit & Londhe^23^, Prahlada & Deka^53^ applied a discrete wavelet transform to decompose time series data into low and high-frequency components. Separate neural network models were then trained for both parts, potentially increasing the accuracy of extreme event prediction^23,53^.

Despite the limited availability of storm data for model training, the XGBoost model performed well and predicted all storms in the unseen test period. Most accuracy-driven 'vanilla' machine learning methods suffer a decline in performance when they encounter a label-imbalanced classification situation^54^. In this experiment, the number of samples in the Storm and No-storm classes was 68 (0.7%) and 9064 (99.3%), respectively. If the model were to predict all the samples as 'no storm,' then the accuracy would be 99%, which is remarkably high. However, failure to predict any storm can lead to severe consequences. The most commonly used evaluation metrics, such as accuracy and precision, were therefore not considered in this study due to their sensitivity to imbalanced data^52^.

Consequently, the model was evaluated using recall, specificity, FPR, FNR, and AUC metrics and provided values of 1, 1, 0, 0, and 1, respectively. These assessment metrics are not sensitive to changes in data distribution and can be used to evaluate classification performance with imbalanced data^52^. Kabir & Ludwig also suggest addressing the problem of imbalanced data by adopting several data resampling techniques before applying XGBoost for classification^55^. More recently, Wang et al. introduced imbalance-XGBoost. The aim of this XGBoost-based Python package is to deal with binary label-imbalanced classification issues by implementing weighted cross-entropy and focal loss functions on XGBoost^54^.

As part of our future work, we plan to implement a multilevel decomposition of data using a discrete wavelet transform. A separate model will then be trained on the low-frequency components (extreme wave height, wave period and wind speed partition) to improve prediction accuracy and predict the characteristics of storm events precisely. To overcome the data imbalance problem, we must also investigate the sampling-based approach of modifying the dataset to balance the class distribution before using it to train the XGBoost classifier. Future research may extend the outcomes of this study to other regions, deep learning model architectures, and hyperparameter tunings.

## Conclusions

This paper considerably expands on past storm prediction research by adopting a unique, integrated strategy that uses both LSTM and XGBoost models to anticipate a wide range of storm characteristics as well as storm occurrence along France's western coast. The detailed investigation reported in this research led to the following conclusions:

- Relevance to Previous Studies: Building on the existing literature that studied the usage of LSTM and XGBoost models in isolation, this study proposes a unique technique that leverages both models in unison. While past work focused largely on specific features of storm prediction, such as storm surges, and approached them as regression issues, the present method blends regression and classification approaches to give a more holistic understanding of storm dynamics. This dual-model technique provides for the thorough prediction of varied storm parameters (temperature, pressure, humidity, wind speed, wave height, and wave period) as well as the prediction of storm occurrence days.
- Performance of the LSTM Model: The LSTM model has shown great accuracy in forecasting temperature and pressure, key elements for understanding and anticipating storm conditions. However, it revealed limits in reliably forecasting factors linked with high variability and extreme circumstances (extreme values), such as wind speed and wave height, particularly during severe storm events. This shows that although the LSTM model is resilient under stable conditions, more refinement and enhancement are required to capture the entire spectrum of storm-induced variabilities.
- Efficiency of the XGBoost Model: The XGBoost model successfully predicted storm occurrences with extraordinary accuracy, indicated by flawless recall and specificity scores. This accuracy is crucial for practical applications in storm forecasting, where the cost of FN (failing to anticipate a storm) can be extraordinarily significant. The performance of the XGBoost model in this situation underlines its promise as a trustworthy tool in operational meteorology.

In essence, this study indicates a big step forward in storm prediction. It not only expands the scope of forecasted storm features but also combines the capabilities of LSTM and XGBoost, opening up new paths for increasing storm preparation and risk reduction measures. The method adopted in this study is not only relevant to the western coast of France but also has possibilities for adaption and usage in other places prone to extreme weather events.

## Data Availability

The final dataset analyzed in this study is available on GitFront: https://gitfront.io/r/user-9374464/JTvFdsL1Q3C5/Storm-Prediction/.

## Code availability

Scripts reproducing the experiments of this paper are available at GitFront https://gitfront.io/r/user-9374464/JTvFdsL1Q3C5/Storm-Prediction/.
